# The complete chloroplast genome sequence of *Amorphophallus konjac* (Araceae) from Yunnan, China and its phylogenetic analysis in the family Araceae

**DOI:** 10.1080/23802359.2023.2300471

**Published:** 2024-01-08

**Authors:** Lifang Li, Ying Qi, Penghua Gao, Shaowu Yang, Yongteng Zhao, Jianwei Guo, Jiani Liu, Feiyan Huang, Lei Yu

**Affiliations:** Yunnan Urban Agricultural Engineering and Technological Research Center, College of Agronomy, Kunming University, Kunming, China

**Keywords:** Araceae, *amorphophallus konjac* K. Koch ex N.E.Br 1858, chloroplast genome, phylogenetic analysis

## Abstract

This work determined and analyzed the complete chloroplast genome sequence of *Amorphophallus konjac* K. Koch ex N.E.Br 1858 from Yunnan, China. The genome size was 167,470 bp, of which contains a large single-copy region (LSC 93,443 bp), a small single-copy region (SSC 21,575 bp), and a pair of inverted repeat regions (IR 26,226 bp). The chloroplast genome has 131 genes, including 86 protein-coding genes, 37 tRNAs, and eight rRNAs. A previous study reported deletion of *acc*D, *psb*E, and *trn*G-GCC genes in the *A. konjac* chloroplast genome. Our study supports the conservative structure of *A. konjac* and does not support the gene deletion mentioned above. Phylogenetic analysis indicated that *A. konjac* shares a close relationship with another *A. konjac* (collected from Guizhou) and *A. titanium* by forming a clade in the genus *Amorphophallus*. Our results provide some useful information to the evolution of the family Araceae.

## Introduction

*Amorphophallus konjac* K. Koch ex N.E.Br 1858 is a perennial, herbaceous monocot that is mainly distributed in Southeast Asia and Africa and belongs to the genus *Amorphophallus* (Chua et al. 2010; Hu et al. 2019). *A. konjac* also is an important economic crop widely used in health products and biomaterials because of its tuber contains a large amount of konjac glucomannan (KGM) (Gao et al. 2022). The high quality and purity of KGM obtained from *A. konjac* makes this species the first most cultivated *Amophophallus* species in China, especially in Yunnan, Guizhou, and Hubei (Sun et al. 2023). Previously, two chloroplast genomes of *A. konjac* from different regions have been reported (Hu et al. 2019; Liu et al. 2019). Moreover, a chloroplast genome of *A. konjac* reported by Liu et al. (2019) found that some genes deletion, including deletion of *acc*D, *psb*E, and *trn*G-GCC. This phenomenon may be attributed to errors in either the assembly, or the annotations, or both (Henriquez et al. 2021). Yunnan province is one of the largest plantation areas of *A. konjac* in China and has many endemic local *A. konjac* resources. The local *A. konjac* resources of Fuyuan are the most representative local varieties in Yunnan, and also an economically important crop for rural revitalization in Yunnan Province. To date, the chloroplast genome of local *A. konjac* resources of Fuyuan prefecture is still lacking. To develop and utilize local *A. konjac* resources of Fuyuan prefecture in Yunnan, and to compare the chloroplast of *A. konjac* from different distributed regions, we sequenced the complete chloroplast genome of *A. konjac* from Yunan, China.

## Materials and methods

**Figure 1.**
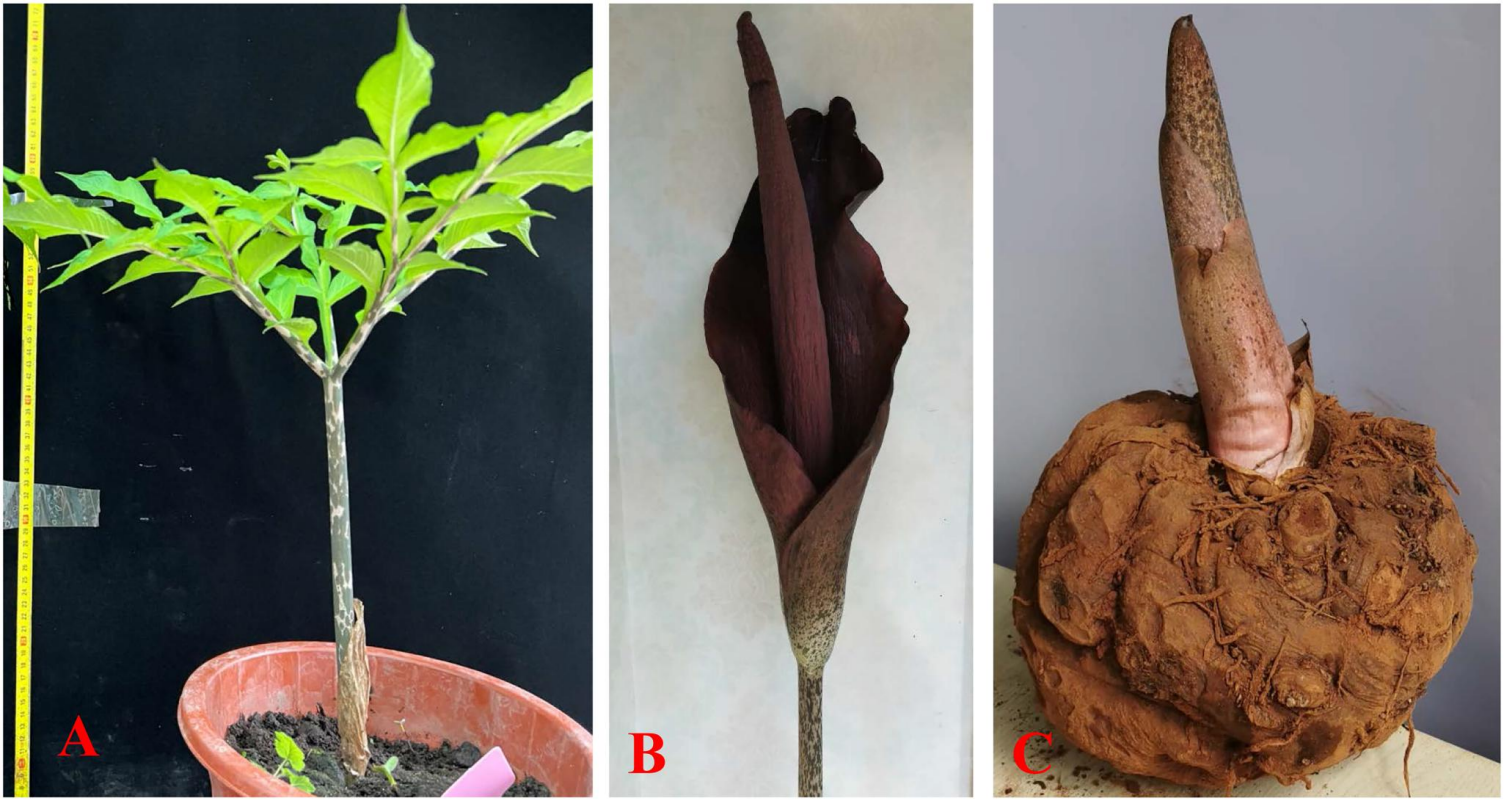
Species reference image of *Amorphophallus konjac*. (A) Plant of *A. konjac*. (B) flower of *A. konjac*. (C) corm of *A. konjac*. These photos were taken by Penghua Gao and Yu Lei at Kunming city, Yunnan Province, China. It is a perennial herb with dark purple flowers and green palmate compound leaves. The petiole has the background color dirty cream-colored, often nearly entirely covered by blackish green spots.

The corms of Fuyuan local *A. konjac* resources were cultivated in Kunming University. Then, the fresh leaves of *A. konjac* were collected from the Konjac Genetic Resources Garden of Kunming University, Yunnan Province (Figure 1, 24.97406°N, 102.79605°E), and a specimen was deposited at the Herbarium of Yunnan Urban Agricultural Engineering and Technological Research Center (Kunming, China, Website: https://www.kmu.edu.cn/zzjg/kyjg.htm, Li-Fang Li, lilf0215@163.com) under the voucher number HMY20230616. Total genomic DNA was extracted and sequenced on the Illumina HiSeq 2500 platform (Illumina, CA, USA). The quality of the short reads was checked with FastQC. *De novo* assembly was conducted using metaSPAdes (Sun et al. 2022). The complete chloroplast genome of *A. konjac* (MK611803) from Guizhou was used as a reference. The assembled chloroplast genome was annotated by CPGAVAS2 (Shi et al. 2019), whereas the tRNA genes were further confirmed by tRNAscan-SE v.2.0 (Chan et al. 2021). MAFFT v7.419 was employed to align the chloroplast genome sequence of three *Amorphophallus* species and adjusted manually with BioEdit (Katoh et al. 2013). Then, DnaSP v6 software (Rozas et al. 2017) was used to identify high variation regions with a sliding window analysis with a window length of 600 bp and a step size of 200 bp. Furthermore, the maps of the annotated chloroplast genome and cis-splicing/trans-splicing genes of *A. konjac*were processed by CPGview (Liu et al. 2023).

The complete chloroplast genomes of 26 Araceae species were downloaded from GenBank and aligned using MAFFT v7. Then, the maximum likelihood (ML) method was used to construct the phylogenetic tree by using IQ-TREE v1.6.12 (Nguyen et al. 2015) with 1000 bootstrap replicates. The *Atractylodes chinensis* (MT834519) and *Iris missouriensis* as outgroups.

## Results

**Figure 2.**
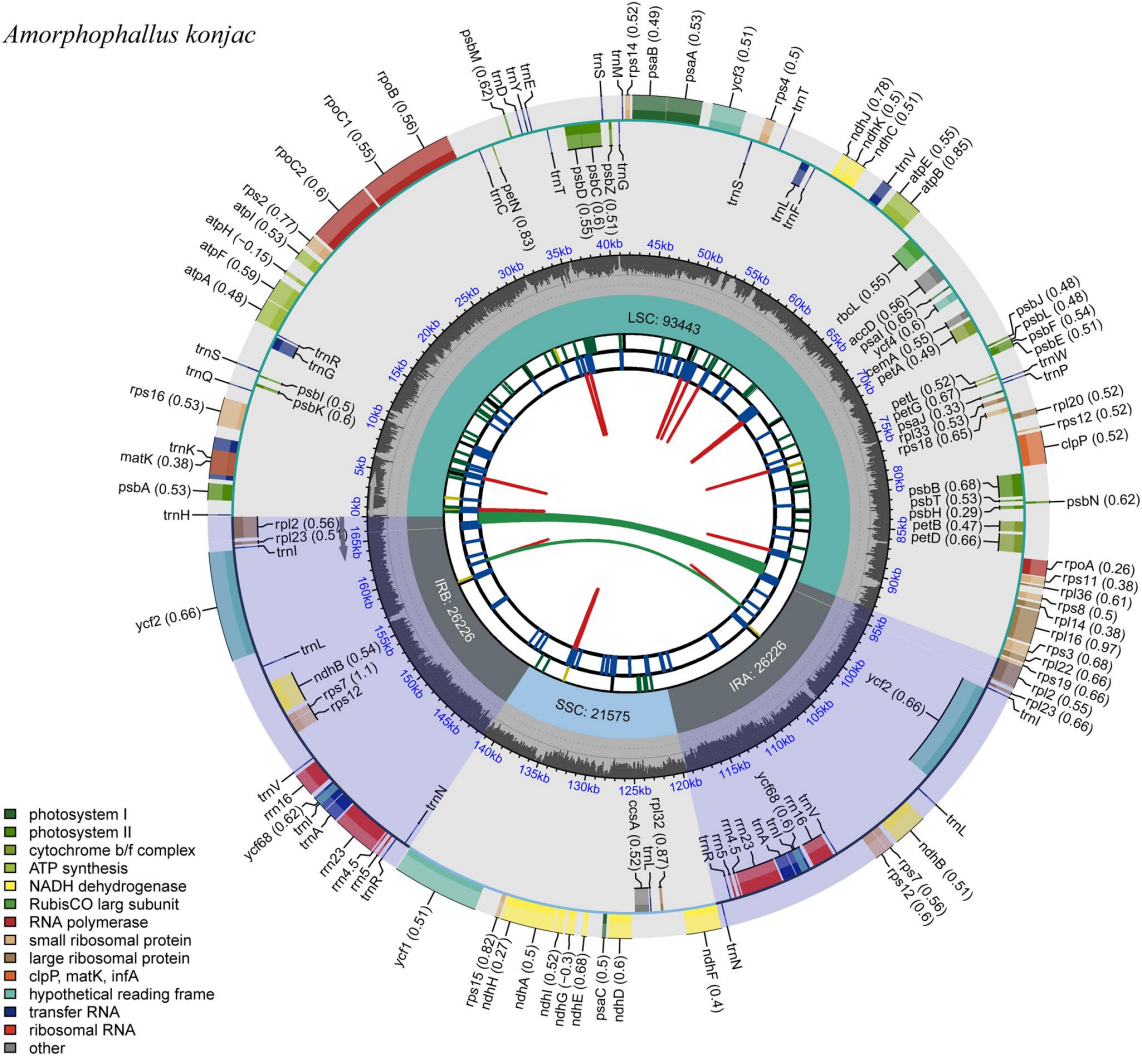
The chloroplast genome map of *Amorphophallus konjac*. From the center, the first track shows the dispersed repeats, including direct repeats (red) and palindromic repeats (green). The second and third tracks show the long and short tandem repeats, respectively. The small single-copy (SSC), inverted repeat (IRa and IRb), and large single-copy (LSC) regions are shown on the fourth track. The GC content along the genome is plotted in the fifth track. The genes are shown on the sixth track.

The chloroplast genome of *A. konjac* was 167,470 bp in length, like other reported plant chloroplast genomes, the genome has a conserved quadripartite structure, including a large single copy (LSC, 93,443 bp), a small single copy (SSC, 21,575 bp) and a pair of inverted repeats (IRa and IRb, 26,226 bp) (Table S1, Figure 2). Finally, the obtained chloroplast genome sequence was constructed with a 4500 coverage depth (Figure S1) and submitted to the NCBI database (Accession number: OR438675). Additionally, the overall GC content in complete chloroplast genomes was 35.4%, and the GC contents of the LSC, SSC, IRa, and IRb regions were 35.4%, 29.7%, 41.5%, and 41.5%, respectively (Table S1). The genome encodes a total of 131 genes, including 86 protein-coding genes, 37 transfer-RNA genes (tRNA), and 8 ribosomal RNA genes (rRNA) (Table S1). Among them, six tRNA genes (*trn*A-UGC, *trn*G-UCC, *trn*I-GAU, *trn*K-UUU, *trn*L-UAA, *trn*V-UAC) and 13 protein-coding genes (*pet*B, *pet*D, *atp*F, *ndh*A, *ndh*B, *rps*12, *rps*16, *rpl*16, *atp*F, *rpo*C1, *rpl*2, *acc*D, *ycf*68) containing one intron and two genes (*ycf* 3, *clp*P) having two introns, whereas all other genes contained one intron (Table S2). Moreover, most of the gene species occurred in a single copy, while 19 gene species occurred in double copies, including all rRNA species, eight tRNA species, and 7 protein-coding species (Table S2). Meanwhile, the chloroplast genome contained 16 cis-splicing genes (Figure S2) and one trans-splicing gene (Figure S3). In addition, the nucleotide variability (Pi) values of *A. konjac* and the closely related species *A. konjac* from Guizhou and *A.titanum* were analyzed. The pi value of nucleotide diversity ranged from 0 to 0.13, and a total of seven highly variable sites were found, including *rps*16 (0.13), *ycf*1 (0.068), *trn*S-GCU (0.055), *ycf*4*-cem*A (0.053), *trn*G-UCC (0.043), *trn*C-GCA*-pet*N (0.422) and *psb*D (0.036) (Figure S4).

**Figure 3.**
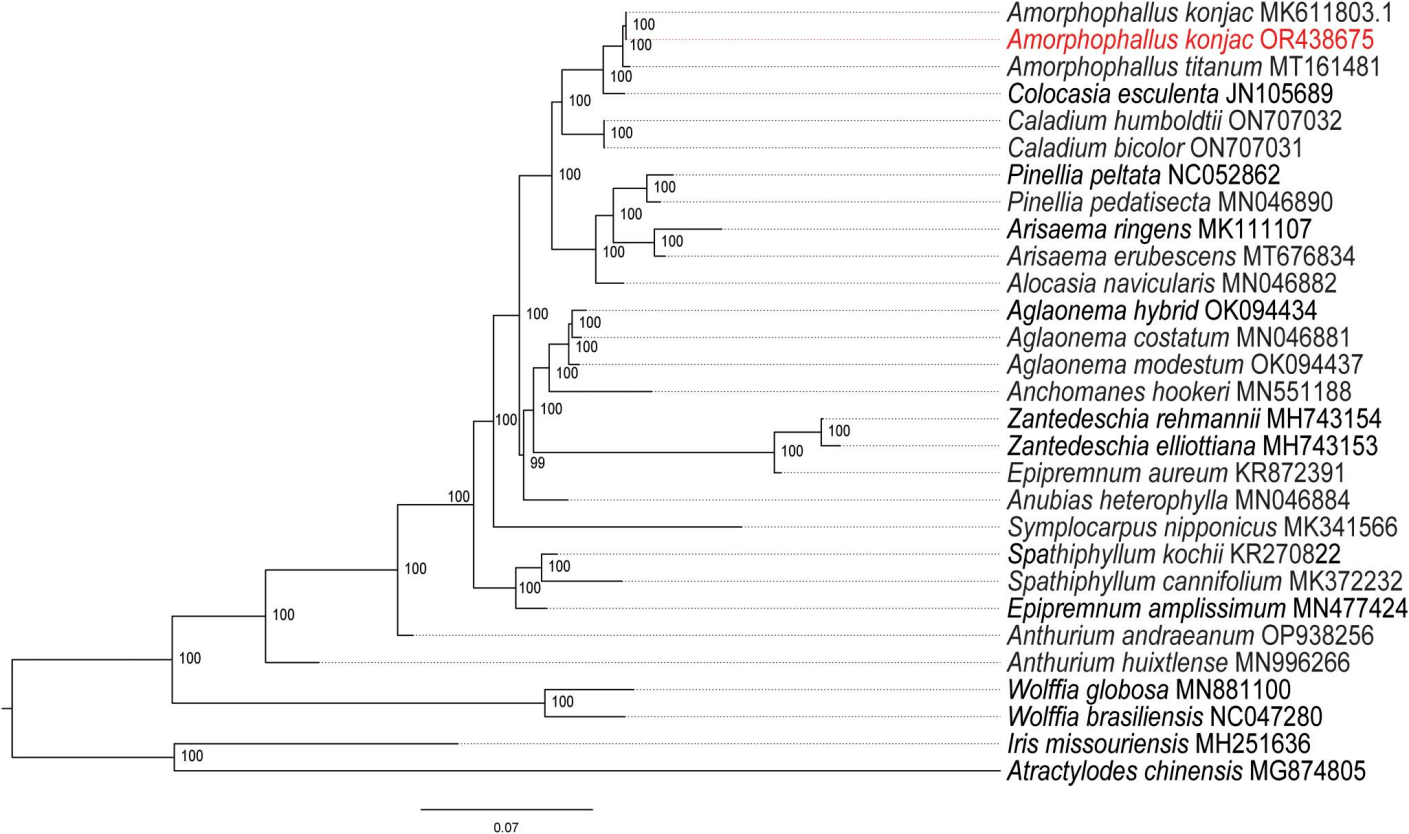
Phylogenomic tree of *Amorphophallus konjac* (in red) and 28 species constructed using maximum-likelihood method based on complete chloroplast genome sequences. *Iris missouriensis* and *Atractylodes chinensis* are used as an outgroups. The best-fit model according to the bayesian information criterion (BIC) was TVM + F+R6. Numbers at each node represent the bootstrap values for 1000 replicates. The following sequences were used: *Amorphophallus konjac* MK611803.1 (Hu et al. 2019), *Amorphophallus titanum* MT161481 (Henriquez et al. 2021), *Colocasia esculenta* JN105689 (Ahmed et al. 2012), *Caladium humboldtii* ON707032 (Ye et al. 2022), *Caladium bicolor* ON707031 (Ye et al. 2022), *Pinellia peltata* NC052862 (unpublished), *Pinellia pedatisecta* MN046890 (Henriquez, Ahmed, et al. 2020), *Arisaema ringens* MK111107 (unpublished), *Arisaema erubescens* MT676834 (Zhang et al. 2020), *alocasia navicularis* MN046882 (Henriquez, Ahmed, et al. 2020), *Aglaonema hybrid* OK094434 (Li et al. 2022), *Aglaonema costatum* MN046881 (Henriquez, Ahmed, et al. 2020), *Aglaonema modestum* OK094437 (Li et al. 2022), *anchomanes hookeri* MN551188 (Henriquez, Ahmed, et al. 2020), *Zantedeschia rehmannii* MH743154 (He et al. 2020), *Zantedeschia elliottiana* MH743153 (He et al. 2020), *Epipremnum aureum* KR872391 (Tian et al. 2018), *anubias heterophylla* MN046884 (Henriquez, Ahmed, et al. 2020), *symplocarpus nipponicus* MK341566 (Kim et al. 2019), *Spathiphyllum kochii* KR270822 (Han et al. 2016), *Spathiphyllum cannifolium* MK372232 (Liu et al. 2019), *Epipremnum amplissimum* MN477424(unpublished), *Anthurium andraeanum* OP938256 (Wan et al. 2023), *Anthurium huixtlense* MN996266 (Henriquez, Mehmood, et al. 2020),*Wolffia globosa* MN881100 (unpublished), *Wolffia brasiliensis* NC047280 (unpublished), *Iris missouriensis* MH251636 (unpublished), *Atractylodes chinensis* MG874805 (Wang et al. 2020).

To analyze the phylogenetic relationship of Araceae species, we constructed a phylogenetic tree using whole chloroplast genome sequences. The maximum likelihood (ML) tree showed that species in the genus *Amorphophallus* were grouped into a monophyletic group with high support value, and *A. konjac* is a sister to *A. konjac* (MK611803) which was collected in Guizhou, China. In addition, *A. konjac* was clustered with *A. titanum* into a subclade with 100% supported value and the genus *Amorphophallus* was sister to the genus *Colocasia*, *Caladium* and *Pinellia* (Figure 3).

## Discussion and conclusion

In the current study, the chloroplast genome of *A. konjac* was 167,470 bp in length which was similar to the published one (*A. konjac* from Wuhan, SRR7938681, 167, 424 bp) (Table S1). On the contrary, the chloroplast genome assembled in this study was 1548 bp longer than that of another published one (*A. konjac* MK611803, 161,647 bp) mainly due to the expansion of the LSC and IR regions (Table S1). Furthermore, the overall GC content (35.63%) and genome quadripartite structure were highly similar to those in other Araceae chloroplast genomes, including *A. konjac* and *A. titanium* (Hu et al. 2019; Henriquez et al. 2021). Significantly, a previous study reported deletion of *acc*D, *psb*E and *trn*G-GCC genes in *A. konjac* from Wuhan (Liu et al. 2019). Henriquez et al. (2021) speculate that this phenomenon may be caused by either assembly errors, or annotation errors, or both. The Organellar Genome Annotator (DOGMA) annotation software they use is prone to errors (Henriquez et al. 2021). Our study shows similar gene content to previous reports in aroids as well as to *A. konjac* (MK611803.1) and *A. titanium* (MT161481), which supports the conservative structure of chloroplast genomes in the *Amorphophallus* species and does not support gene deletion mentioned above. Our phylogeny showed that *A. konjac* was closely related to *A. konjac* (MK611803.1) and *A. titanium* (MT161481), which is congruent with the previous study (Henriquez et al. 2021). Finally, this study improve our understanding of the characteristics of the chloroplast genome of *A. konjac* and are valuable for future breeding and research efforts.

## Supplementary Material

Supplemental Material

Supplemental Material

Supplemental Material

## Data Availability

The assembled chloroplast genome sequence data that support the findings of this study are openly available in GenBank of NCBI (https://www.ncbi.nlm.nih.gov/) under the accession number of OR438675. The associated BioProject, SRA, and Bio-Sample numbers are PRJNA1008633, SRR25741018, and SAMN37123742, respectively.
