# Supplementary material for: The complete chloroplast genome sequence of Amorphophallus konjac (Araceae) from Yunnan, China and its phylogenetic analysis in the family Araceae
Source: Mitochondrial DNA B Resour. 2024 Jan 8;9(1):41–5. doi: 10.1080/23802359.2023.2300471 (PMC10776074; doi:10.1080/23802359.2023.2300471)
Supplement: Supplemental Material [file TMDN_A_2300471_SM8716.docx]

Table S1 Summary of the sequencing data for the four *Amorphophallus* species.

|  | *A. konjac* | *A*. *konjac* | *A. konjac* | *A.titanum* |
| --- | --- | --- | --- | --- |
| Region | Longquan  Mountain, Guizhou | Wuhan, Hubei | Kunning, Yunnan | Sumatra, Indonesia |
| total (bp) | 161,647 | 167,424 | 167,470 | 176,835 |
| inverted repeat regions(IRa) | 25,722 | 25973 | 26226 | 32,708 |
| inverted repeat regions(IRb) | 25,722 | 25973 | 26226 | 32,708 |
| a large single copy region(LSC) | 90,006 bp | 92660 | 93443 | 95,475 |
| a small copy region (SSC) | 20,197 bp | 22839 | 21575 | 15,944 |
| total GC% | 42.30% | 35.39 | 35.4 | 34.5 |
| IRa GC% | - | - | 41.5% | 38.6 |
| IRb GC% | - | - | 41.5% | 38.6 |
| LSC GC % | - | - | 33.20% | 32.7 |
| SSC GC% | - | - | 29.70% | 28.5 |
| number of genes | 131 | 111 | 131 |  |
| coding-protein | 86 | 78 | 86 | 85 |
| rRNA | 8 | 4 | 8 | 8 |
| tRNA | 37 | 29 | 37 | 37 |
| Gene Bank | MK611803 | SRR7938681 | OR438675 | MT161481 |
