# Supplementary material for: The complete chloroplast genome sequence of Amorphophallus konjac (Araceae) from Yunnan, China and its phylogenetic analysis in the family Araceae
Source: Mitochondrial DNA B Resour. 2024 Jan 8;9(1):41–5. doi: 10.1080/23802359.2023.2300471 (PMC10776074; doi:10.1080/23802359.2023.2300471)
Supplement: Supplemental Material [file TMDN_A_2300471_SM8714.docx]

Supplemental material


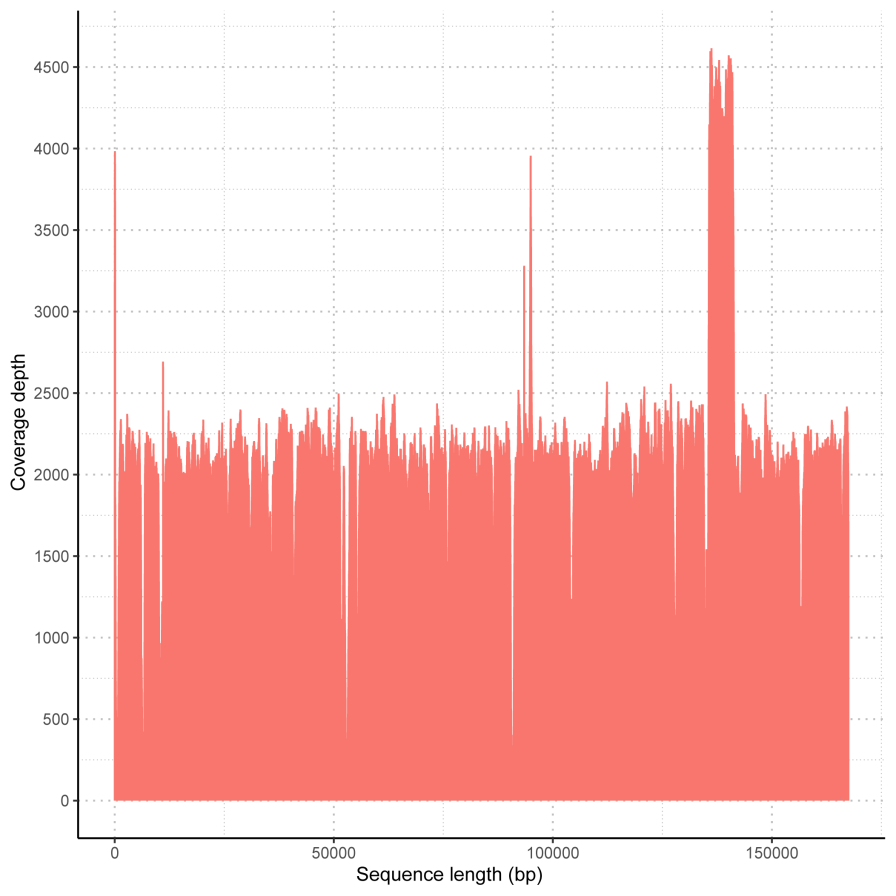


Figure S1. Coverage depth figure of the *Amorphophallus konjac* chloroplast genome. The horizontal coordinate is the base of the chloroplast genome and the vertical coordinate is the depth of sequencing corresponding to that base.


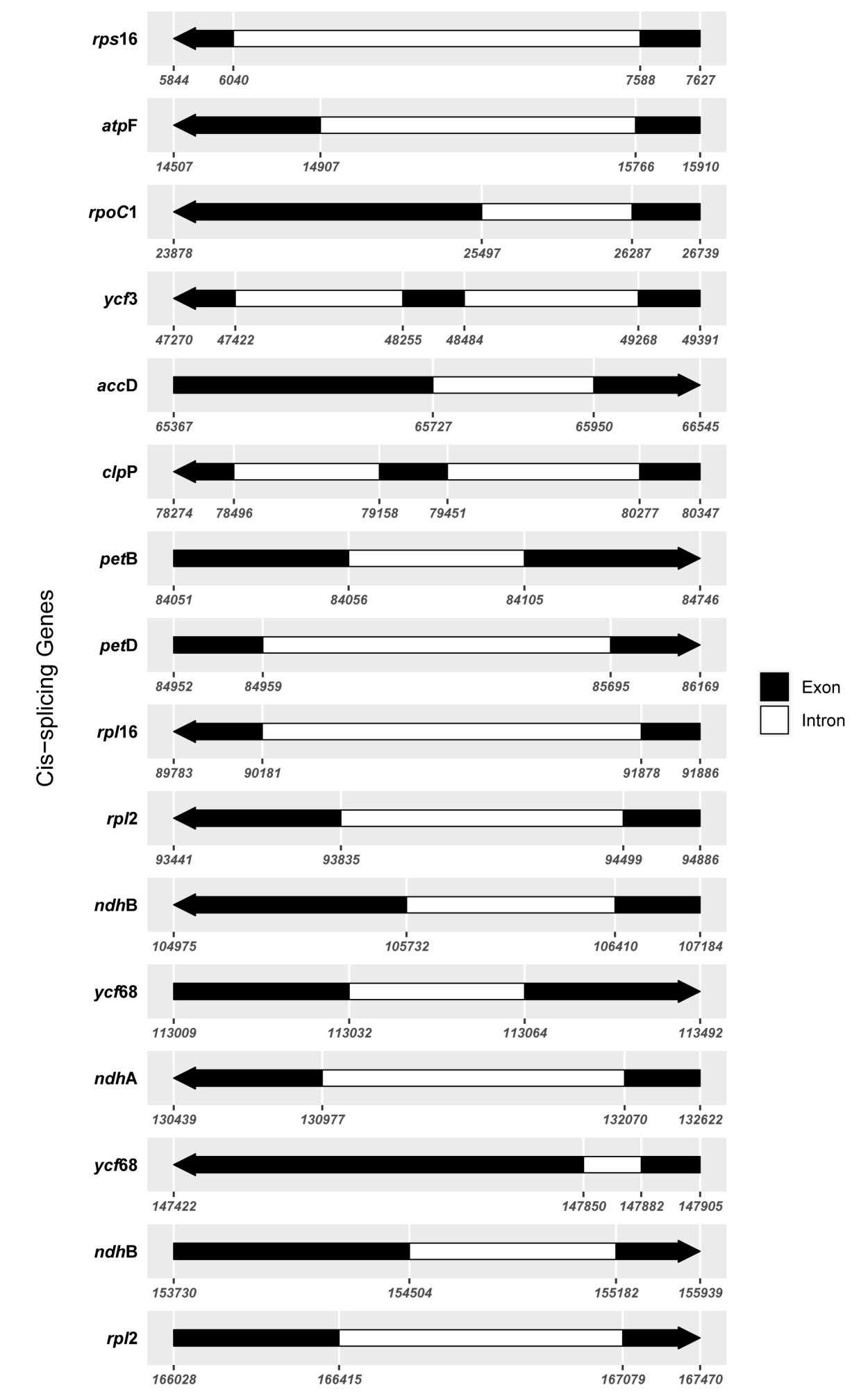


Figure S2. The map of the cis-splicing genes in the *A. konjac* chloroplast genome.


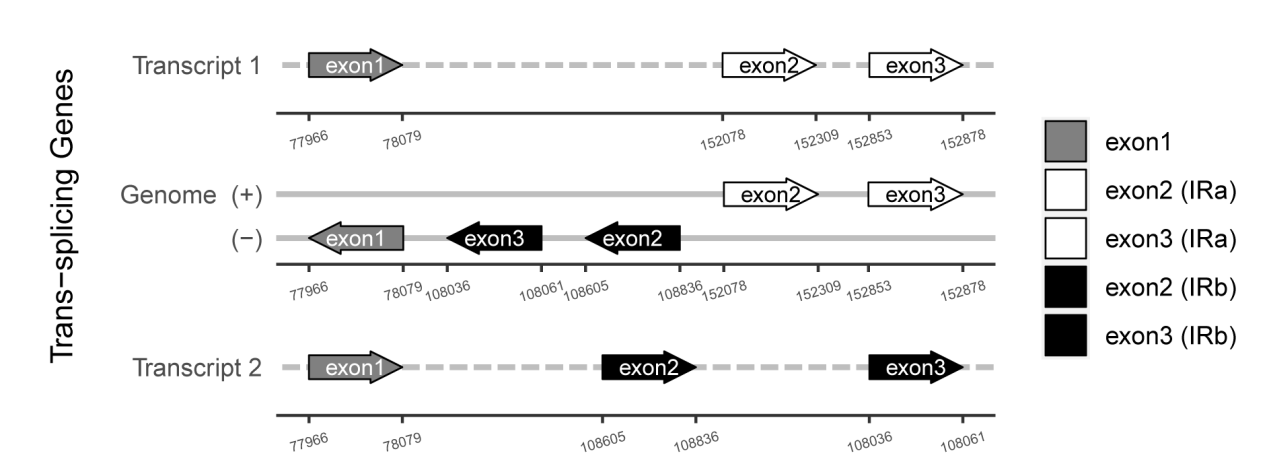


Figure S3. The map of the trans-splicing gene *rps*12 in the *A. konjac* chloroplast genome.


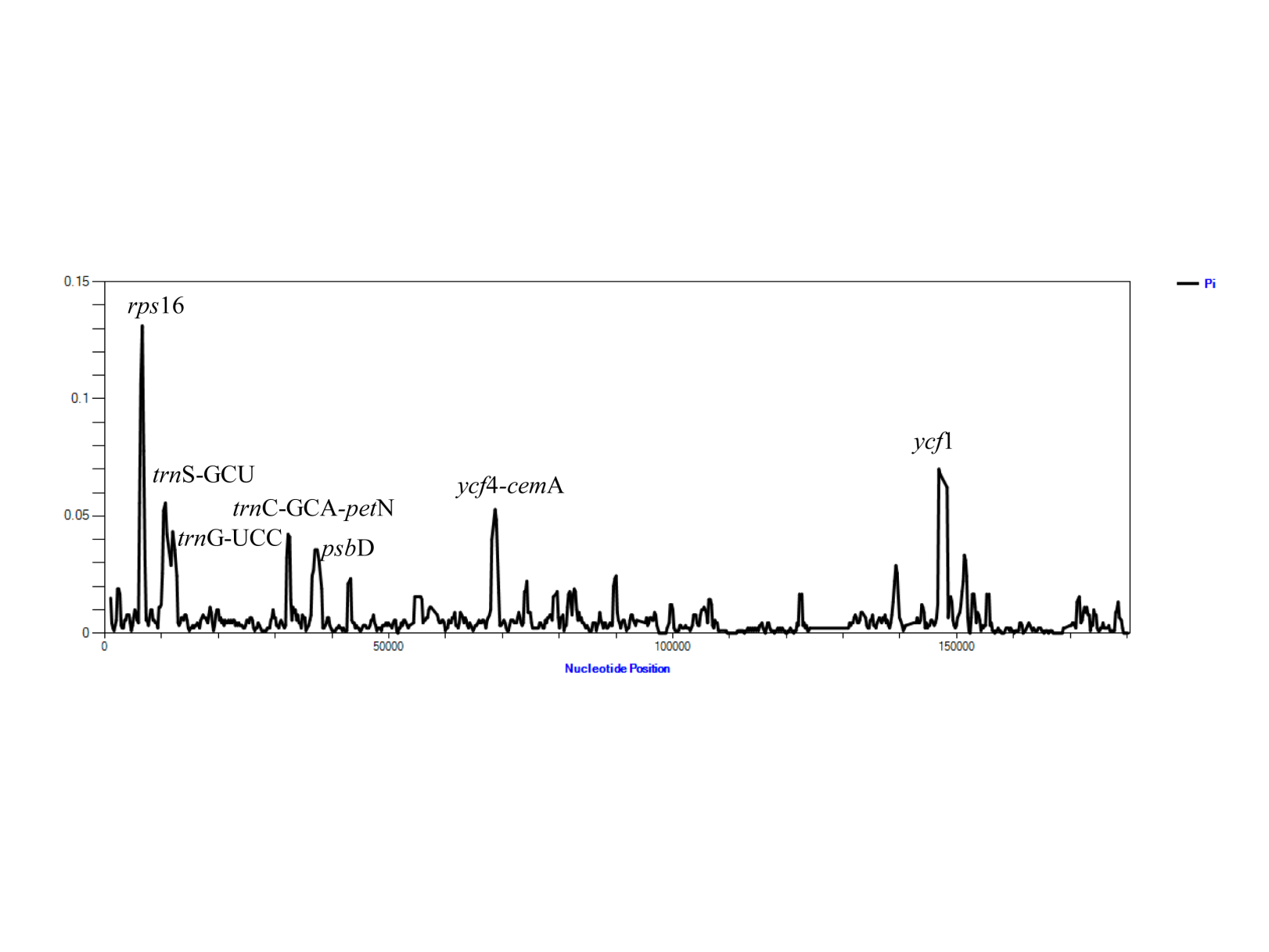


Figure S4. Nucleotide diversity (Pi) comparing the chloroplast genome sequences of the three *Amorphophallus* species using sliding window analysis. Window size: 600 bp, step size: 200 bp. The Y-axis shows the pi values; the X-axis shows the position of the midpoint.
