# Supplementary material for: The complete chloroplast genome sequence of Amorphophallus konjac (Araceae) from Yunnan, China and its phylogenetic analysis in the family Araceae
Source: Mitochondrial DNA B Resour. 2024 Jan 8;9(1):41–5. doi: 10.1080/23802359.2023.2300471 (PMC10776074; doi:10.1080/23802359.2023.2300471)
Supplement: Supplemental Material [file TMDN_A_2300471_SM8713.docx]

Table S2 Genes in *Amorphophallus konjac* chloroplast genome

| Funcation | Gene Group | List of genes |
| --- | --- | --- |
|  | Subunits of photosystem I | *psa*A, *psa*B, *psa*C, *psa*I, *psa*J |
|  | Subunits of photosystem II | *psb*A, *psb*B, *psb*C(2), *psb*D(2), *psb*E |
|  |  | *psb*F, *psb*H, *psb*I, *psb*J, *psb*K, |
|  |  | *psb*L, *psb*M(2), *psb*N, *psb*T, *psb*Z(2) |
| Photosynthesis | Subunits of cytochrome b/f complex | *pet*A, *pet*B*, *pet*D*, *pet*G, *pet*N, *pet*L, |
|  | Subunits of ATP synthase | *atp*A, *atp*B, *atp*E, *atp*F*, *atp*H, *atp*I |
|  | Subunits of NADH-dehydrogenase | *ndh*A*, *ndh*B*(2), *ndh*C, *ndh*D, *ndh*E, |
|  |  | *ndh*F, *ndh*G, *ndh*H, *ndh*I, *ndh*J, *ndh*K |
|  | RubisCO large subunit | *rbc*L |
|  | Small subunit of ribosome | *rps*11, *rps*12*(2), *rps*14, *rps*15, *rps*16* |
|  |  | *rps*18, *rps*19, *rps*2, *rps*3, *rps*4, *rps*7(2), *rps*8 |
|  | Large subunit of ribosome | *rpl*2*(2), *rpl*14, *rpl*16*, *rpl*20, *rpl*22, |
|  |  | *rpl*23(2), *rpl*32, *rpl*33, *rpl*36 |
|  | DNA-dependent RNA polymerase | *rpo*A, *rpo*B, *rpo*C1*, *rpo*C2 |
|  | Ribosomal RNA genes | *rrn*16(2), *rrn*23(2), *rrn*4.5(2), *rrn*5(2) |
|  | Transfer RNA genes | *trn*A-UGC (2)*, *trn*C-GCA, *trn*D-GUC, *trn*E-UUC, *trn*F-GAA, |
|  |  | *trn*G-GCC, *trn*G-UCC*, *trn*H, *trn*I-CAU(2), *trn*I-GAU(2)* , |
|  |  | *trn*K-UUU*, *trn*L-UAA*, *trn*L-CAA(2), *trn*L-UAG, *trn*N-GUU(2) |
| Self-replication |  | *trn*P-UGG, *trn*Q-UUG, *trn*R-UCU, *trn*R-ACG (2), *trn*S-GCU, |
|  |  | *trn*S-UGA, *trn*S-GGA, *trn*T-UGU, *trn*T-GGU, *trn*V-UAC* |
|  |  | *trn*V-GAC(2), *trn*W-CCA, *trn*Y-GUA, *trn*M-CAU |
|  | Maturase | *mat*K |
|  | Protease | *clp*P** |
| other genes | Envelope membrane protein | *cem*A |
|  | Acetyl-CoA carboxylase | *acc*D* |
|  | c-type cytochrome synthesis gene | *ccs*A |
| Genes of unknown function | hypothetical chloroplast reading frames(ycf) | *ycf*1, *ycf*2 (2), *ycf*3**, *ycf*4, *ycf*68(2)* |

Note: *: one intron; **: two intron.
